# A Treatise on Surgery, Its Principles and Practice

**Published:** 1889-08

**Authors:** 


### Book Reviews.

A Treatise on Surgery, its Principles and Practice, by T. Holmes, M. A. Cantab, with four hundred and twenty-eight illustrations.

Fifth edition, edited by T. Pickering Pick, Surgeon to and lecturer on Surgery at St. George's Hospital, Senior Surgeon Victoria Hospital for Children; member of the Court of Examiners Royal College of Surgeons, England. Lea Brothers & Co., Philadelphia, 1889.

This excellent work on Surgery is too well known to our readers to require a critical or detailed review. The general arrangement of the work is the same as in former editions, though many of the chapters have been carefully rewritten and the whole work thoroughly revised so as to bring it in most respects fully up to the standard of modern surgery. The chapter on Inflammation is exceedingly clear and concise, up with the times in every respect, except in the minor details of antiseptic treatment, which are hardly sufficiently comprehensive to satisfy modern enthusiasts on that line.

The chapters on Bones and Joints, Tumors, Abdominal Surgery and Intestinal Obstruction, and Diseases of the Breasts have been thoroughly revised and rewritten so as to include our latest knowledge and best methods of treatment.

The work as a whole is one of the very best text-books for students and practitioners who have not the time to wade through the exhaustive systems and encyclopædias of surgery.

